# Perception of the ethical acceptability of live prey feeding to aquatic species kept in captivity

**DOI:** 10.1371/journal.pone.0216777

**Published:** 2019-08-22

**Authors:** Lucy Marshall, Wanda D. McCormick, Gavan M. Cooke

**Affiliations:** 1 Faculty of Health Sciences, University of Bristol, Langford Veterinary School, Bristol, United Kingdom; 2 Faculty of Life Sciences, Anglia Ruskin University, Cambridge, United Kingdom; 3 Faculty of Health & Society, University of Northampton, Northampton, United Kingdom; University of Sussex, UNITED KINGDOM

## Abstract

Previous research into public perceptions of live prey feeding has been focused on terrestrial animals. The reasons for this likely relate to the difficulty humans have in being compassionate to animals who are phylogenetically distantly related. In order to test these assumptions, the general public (two groups; one who had just visited an aquarium; and one group who had just visited a zoo), aquarium professionals in the UK/US and terrestrial zoo animal professionals (UK) were investigated to see how they would differ in their responses when asked about feeding various live aquatic animals to one another. Likert based surveys were used to obtain data face to face and via online social media. Demographics in previous research identified a lower acceptance of live prey feeding by females, however in aquatic animals this was not reflected. Instead, separations in perception were seen to exist between participants dependent on whether they had just visited a zoo or aquarium, or worked with animals.

## Introduction

Research into public perception of live prey feeding (whether it involves invertebrates or vertebrates as either the prey or predator) has, until now, been focused entirely on terrestrial animals [1, 2]. This research bias is potentially due to a natural tendency to focus more on terrestrial species which elicit a higher emotional attachment [3, 4]. The greater acceptance of the existence of affective states in terrestrial mammals, based on a closer phylogenetic relatedness [5], could also have contributed to the lack of research in this area. Regardless of the reasons, even charismatic aquatic species (such as cetaceans and cephalopods) are often less understood by the public. For example, Barney [6] found public knowledge of dolphins was poor, and opinion was largely based on a person’s emotional and empathetic response rather than the widely available educational information on these animals. This empathy extends even less towards fish (i.e. teleosts) as, despite also being aquatic vertebrates, they are even further removed from humans, not only phylogenetically but also with regards to physical and behavioral similarity [7]. The lack of research into public perception of live prey feeding in fish specifically could be due to a lack of wide-scale understanding of how fish perceive the world. Where it can be assumed that a tiger would suffer behavioral and digestive abnormalities from not hunting live prey [8], the effects this would have on a fish are less well understood by many.

### What capacity do invertebrates and fish have to suffer?

Until relatively recently it was assumed that the absence of a neocortex in invertebrates meant that they could neither feel pain nor comprehend the world past simple internal and external cues [9], but relied on the simplest forms of cognitive processes [10]. This has since been disputed [11, 12, 13] and it has been argued that the neocortex is not indicative of the ability to suffer if analogous structures are present; for example, macaques have no prefrontal cortex yet the presence of subcortical and cortical structures allow them to efficiently problem solve with a potential awareness of their memory ability [14, 15]. Sneddon [13] found that when testing behaviour changes following exposure to noxious stimuli in trout, it resulted in decreased feeding motivation, rocking whilst on substrate surface, and rubbing their snouts on tank walls, indicating aversive and abnormal behavioral reactions related to pain [15]. Studies in cephalopods (molluscs) [16] and decapod crustaceans (i.e. shrimps, crabs) [17] have observed an avoidance of stimuli that could be associated with pain.

The concept of suffering is not merely restricted to pain but also involves the assessment of cognitive ability when considering the impact of behavioral deprivation. Several species of fish have exhibited complex learning behavior, such as the ability to generate internal map-like representations; seen by Aronson [18] in a rock pool gobiid fish who relied on knowledge of escape routes and topography. Observational learning can even be seen in species such as fighting fish, who will observe victors of previous fights and avoid conflicts with them subsequently [19]. Examples exist of both aquatic vertebrates [20] and cephalopods [21] which have exhibited tool use and the ability to modify their behavior to achieve a more beneficial outcome, suggesting a cognitive ability similar to that of terrestrial vertebrates [20]. Feld *et al*. [22] recognized an advanced cognitive ability in decapod crustaceans, whereby information could be stored for several days and complex learning was displayed. This was supported by studies into crabs who consistently avoided a structure similar to where they had previously received a ‘painful’ electric shock [23, 17].

### Is live prey feeding necessary?

Live prey feeding to animals kept in captivity is seen as necessary by some to promote behaviours that occur naturally in the wild [8] and therefore may have beneficial impacts on the animals’ behavior, general health and lifespan [24]. Live prey feeding may, however, may be detrimental to the wellbeing of the predator due to injury risk when hunting and killing [25] and energy expenditure [26] in an unnatural and/or finite enclosure, cage or tank. A key argument by opponents to live feeding is the suggestion that well-designed environmental enrichment can essentially replace the behavioral opportunities that would otherwise be lost. For example, Quirke *et al*. [27] documented comparable speeds attained by a cheetah exposed to a ‘cheetah run’ device whereby a lure is followed to simulate hunting. However, not all attempts at enrichment are successful in recreating experiences afforded by the presence of live prey, as demonstrated by Skibiel *et al* [28] in their provision of raw bones to captive large felids. A brief review of positive and negative aspects of live prey feeding can be seen in Table 1.

**Table 1:** A brief list of examples of positive and negative aspects of live prey feeding.

Aspect Affected:	‘For’ Live Prey Feeding	References	‘Against’ Live Prey Feeding	References and Species Example
**Health**	Live food is essential for survival	Birds [25] Juvenile seahorses [29, 31]. Snakes [25]. Cephalopods [30, 31].	The process of hunting and killing may cause injury to predator	Snakes [25]. Cuttlefish [32].
	Dental benefits	Big cats [2].		
**Behaviour**	Enrichment and activity having a positive effect on reducing stereotypes and encouraging ‘natural’ behavior	Big cats [32].	Might increase territorial and aggressive behavior in animals less able to catch prey.	Rainbow trout [33].
**Learning required skills**	Parent offspring learning or conspecific social learning necessary for survival following release	Fish [34].		
**Ethics**	Ideal enrichment	Big cats [35].	Inhumane treatment of prey	Mice [25].

Assessments on behavior changes of aquatic animals’ dependent on a live prey diet are few in comparison to terrestrial mammalian studies [36]. Despite fewer studies of the effects in aquatic species there is evidence to justify live prey feeding amongst them. Cuttlefish (i.e. *Sepia officinalis*), for example, exhibit greater growth and survival rates when fed live instead of frozen shrimp [37]. A similar pattern is seen in seahorses; and prohibiting a live prey diet can even have fatal consequences on developing fry [31]. Conversely, this health benefit is lost if the damage caused by hunting prey is significant, which can happen in small tanks (Cooke pers.obs) as some common captive aquatic predators (e.g. cephalopods) damage easily in captivity [31]. Regardless of potential harm, learned predatory behaviour may be a necessary skill for fish to obtain if they were to be re-released for conservation goals [38]. Trout with predatory experience were seen to be significantly more skilled than those without, which had a substantial effect on their growth, mortality, reproduction and health when released [24]. Cox and Pankhurst [39] recognize this as a reluctance of inexperienced trout to feed on novel prey.

## Live prey feeding and legislation

Legislation exists in many countries which describes the circumstances in which live prey feeding would be acceptable and where it would not (S1 Table in supplementary materials). Laws differ across countries and are frequently interpreted in different ways; for example, to ‘minimise suffering’ under the Animal Welfare Act (UK) [40] could be seen as providing a normal stimulation and thereby improving welfare of the predatory species by feeding it live prey, or conversely to avoid using live prey in order to eradicate the prey’s suffering of being eaten alive [25]. In the UK, such circumstances allowing live prey feeding require written justification and ethical review, and only after being advised to do so by a veterinary surgeon. The feeding must then be observed by trained staff, away from public view and the prey must not be left in the enclosure if not eaten [41]. It can be argued that vague language found in legislation around the world can both encourage and forbid the act [25]; for example, to ‘feed appropriately’ and ‘avoid cruelty’ could be seen as evidence to support both opposing sides. Table 2 details legislation on live prey feeding in various countries.

**Table 2:** Legislation regarding the act of live feeding around the world.

Country	Department	Relevant Act/s	What it Means
US	USDA, APHIS and Animal Care	Veterinary Surgeons Act [44] and the Humane Methods of Slaughter Act [45].	Animals must be unconscious before slaughter and may be applied to prey being fed. There is, however, no direct law prohibiting the feeding of live prey.
EU/ UK China	EU Directive 98/58/EC. Often up to member states. DEFRA	Animal Welfare Act [40] and Zoo Licensing Act [43].	Live vertebrate prey is to be discouraged, save for exceptional circumstances where veterinary advice is necessary.
		The Welfare of Farmed Animals [46].	Animals may not be fed anything that could cause them harm.
		European Convention of the Protection of Animals Kept for Farming Purposes (Article 3, 6, 9 and 14) [47].	Applies only to farmed, vertebrate fish. Fish feeding must be appropriate for species and health must be optimal. Prey may cause harm and can be avoided if diet is otherwise suitable. Animals’ food must be appropriate for their physiological and ethological needs in accordance with scientific knowledge, however, no food may be given that could cause unnecessary harm.
		1999/22/EC; Keeping of Wild Animals in Zoos (Article 3) [48]	Animals must be accommodated in conditions that satisfy their biological and conservation requirements, with species specific enrichment.
		Animal Welfare Act [40] (companion, farming, zoos); Animals (Scientific Procedures) Act (ASPA [42]) and the Zoo Licensing Act [43].	The feeding of live, vertebrate prey is to be discouraged, save for exceptional circumstances where veterinary advice is necessary.
	n/a	No relevant laws currently in operation.	No restrictions. Live prey feeding occurs in many institutions around China.
South Africa	NSPCA	Zoo Licensing Act [43].	Only applies to vertebrates, preventing cruelty but without specific mention of live prey feeding.
Australia (state specific)	Australian Capital Territory	Animal Welfare Act [40].	Prohibits causing pain to vertebrates and invertebrates. Would discourage live prey feeding.
Australia (state specific) Russia	New South Wales	Prevention of Cruelty to Animals Act [49].	Prohibits causing pain to vertebrates and invertebrates. Would discourage live prey feeding.
	Queensland	Animal Care and Protection Act [50].	Creates a duty of care applying to vertebrates and some cephalopods. They could not be used as live prey.
	Victoria	Prevention of Cruelty to Animals Act [49].	Protects all vertebrates and adult cephalopods from cruelty. They could not be used as live prey.
	Russian Penal Code	Article 245 [51]	Prohibits cruelty to animals involving death or injury if the deed has been conducted with malicious intent. Would potentially discourage live prey feeding for those reasons.

Opinion based questionnaires have been used to see if visitors of zoos find live prey feeding ethically acceptable [1, 2] The general outcome suggested broad acceptance, however, there are influencing factors. Females are generally less supportive of live prey feeding and frequent visitors of zoos are more likely to disagree with on-show live feeding of animals. This is particularly significant when compared with those who possess higher education [1]. No comparison exists within this study about frequent visitors who also possess a higher education. There was also a species divide, where ‘*live rabbits being fed to tigers*’ was found unacceptable by a higher number of participants compared to the average survey scores [1]. This may be due to a higher emotional attachment to rabbits as they are frequently kept as pets, or the way in which tigers kill and eat them; which may look unpleasant. Considering the species divide it is plausible to assume that live feeding of aquatic animals to one another would be acceptable, however no evidence either way presently exists, and this study aims to fill that gap.

The aim of this study was to explore the perception of live prey feeding to aquatic animals and to see how this varied in accordance to the taxonomic level of the prey and predator (i.e. invertebrate vs vertebrate) and whether feeding was conducted on or off show (i.e. in front of the public or behind closed doors. The responses were also evaluated in relation to the nationality of the respondent and their connection to the captive aquatic industry (with regards to their employment in or visiting of zoos and aquaria). Other relevant demographics, such as gender, were also recorded.

## Methods

Data was collected by means of a questionnaire (see S1 Survey) from 248 participants in the summer of 2017. Participants were selected opportunistically either by following a link in an online forum (Facebook groups for zoo and aquarium professionals), to obtain participants that worked with animals, or personally at Paignton Zoo Environmental Park (Paignton, UK) and Living Coasts Aquarium (Torquay, UK), for members of the public who had just visited either terrestrial animals in a zoo or aquatic animals in an aquarium. Data was collected as participants were leaving the establishments to ensure they had gained appropriate experiences that would set them aside from general members of the public who had not had recent contact with either of these groups.

The questionnaire was similar in all four cases, however when asking those who worked with animals, the question; ‘which type/s of animal do you own?’ was changed to ‘which type/s of animals do you work with?’. The demographics collected (see supplementary materials) allowed us to assign experience of various animals kept professionally into two groups; those who keep aquatic animals and those who do not. As some zoos possess aquaria a narrow focus on what the collection was called was avoided.

It is noted by the researchers that this sample will not represent the population of the UK as there is bias involved; towards those that are able and keen to visit a zoo or aquarium (potentially having more knowledge about animal husbandry due to their interest) and towards those who use social media (which may create an age bias). This has been seen by the exclusion of participants aged 65 years or older due to too small a sample size (n = 7). By using Facebook and sampling participants who have visited a zoo or aquarium there is also likely to be a bias created through access to resources, ignoring a percentage of the population who have access to neither of these things. This could potentially have been accounted for if a control group was put in place, by asking members of the public on a busy high street which is more likely to include a larger demographic.

The questionnaire used a Likert scale with 5 possible answers (e.g. definitely agree, agree, do not know, disagree, strongly disagree). Positive and negative answers were randomly alternated to keep the participants’ attention throughout the form to avoid ‘reverse-scoring’ [54], as were the order of the agreements. Using the scales, participants were asked to respond in relation to seven specific feeding scenarios:

1. The feeding of live fish to shark (in view or away from public view)
2. The feeding of live crabs to cuttlefish (in view or away from public view)
3. The feeding of live fish to another fish (in view or away from public view)
4. The feeding of live fish to cuttlefish (in view or away from public view)
5. The feeding of live shrimp to fish (in view or away from public view)
6. The feeding of live octopus to shark (in view or away from public view)

These feeding scenarios allowed appropriate separation of different taxa and feeding styles that would allow clearer results when comparing any differences in scores. By the inclusion of asking participants for their views on said feeding when in public view, the division between beliefs of how ethical live prey feeding is and whether the public should see it can also be observed separately. The choice of live animals chosen reflects the likely animals found in public aquariums and what they may be fed for nutrition and enrichment (Cooke pers.obs).

An online form was used to ease the processing of data. Once data was in a spreadsheet format, answers were given scores to ease the transmission of data into SPSS v20; so, answers finding live prey feeding ethically acceptable were scored higher (i.e. 5) and answers finding it unacceptable were scored lower (i.e. 1). Demographics were removed if n<10 (e.g. removing participants aged 65 years old or older and any professional not from the UK or US; consisting of 7 participants being removed). Data were analyzed using parametric tests as data met assumptions for normal distribution. Likert data has been analysed this way before [54] as survey data in this form can be seen as interval like in nature and practice.

The questionnaire was vetted by experts at Bristol Zoological Society (UK) and ethically reviewed by the BIAZA Research Committee. Ethical approval was received from the Anglia Ruskin University Biology Department Research Ethics Panel and the study adhered to their data protection standards.

## Results

Table 3 looks at the demographics of the participants so as to understand potential trends in the results.

**Table 3:** Data for demographics from the survey asking the ethical acceptability of feeding live aquatic animals to one another from the public and animal care professionals.

Country	UK	208
	US	36
Source	UK aquarist	71
	US aquarist	36
	UK non-aquarist	53
	Zoo visitor	49
	Aquarium visitor	34
Age Range	18–24	95
	25–34	92
	35–44	25
	45–54	12
	55–64	12
	64+	7
Sex	Not stated	2
	Male	93
	Female	148

There was a statistically significant difference in the survey scores based on the source of the survey responders (e.g. UK aquarium professional etc) MANOVA, *F* 1.646, p = 0.05; Wilk’s Λ = 0.661. No statistical difference was found between sex or age.

**Fig 1:**
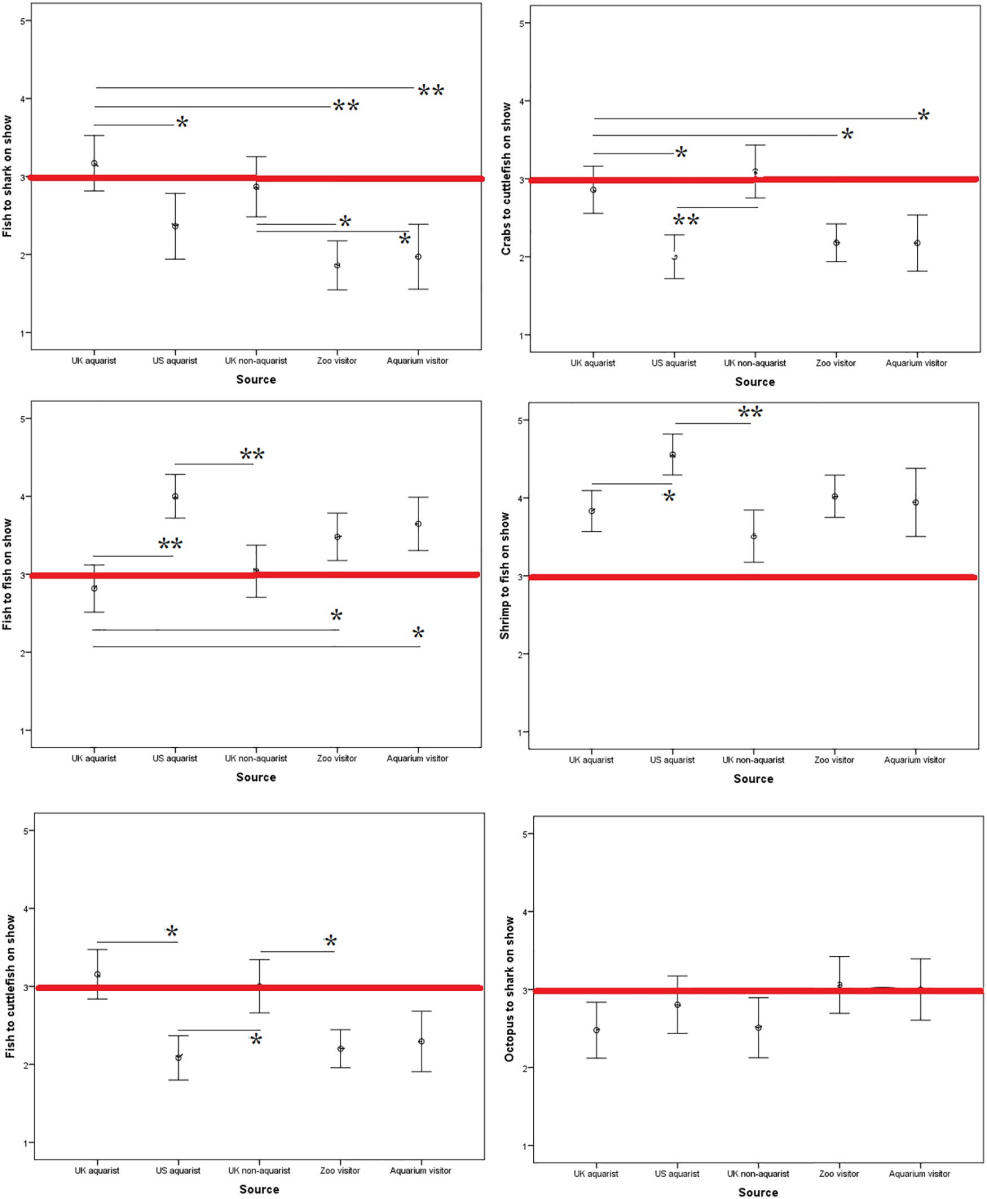
Mean survey scores by source (e.g. UK aquarist etc) for all 12 questions asked regarding the acceptability of feeding various live aquatic animals to one another ‘on show’, i.e. potentially in view of the public. Likert scale (y-axis) ranged from 1 (least acceptable) to 5 (most acceptable), after recoding. The red line indicates the middle available score (i.e. ‘unsure’). Therefore, scores above the red line indicate that the practice is considered acceptable. * = p = <0.05 ** = p = <0.001.

Table 4 shows frequent statistical levels of significance between the variables that are compared further below in Fig 1, grouping the variables by the participants demographics.

**Table 4:** Test of between subject effects for comparisons within the survey responses from with Source (e.g. UK aquarium professional etc). Degrees of freedom equal to 4 for all comparisons. Statistical significance was calculated using Bonferoni corrected ANOVAs and Turkey post hoc tests.

Dependant variable	F	Sig.
Crab to cuttlefish on show	2.580	0.039
Fish to shark on show	2.977	0.020
Fish to fish on show	2.662	0.089
Shrimp to fish on show	0.365	0.833
Fish to cuttlefish on show	2.149	0.076
Octopus to shark on show	0.358	0.839
Fish to shark off show	3.371	0.011
Crabs to cuttle fish off show	2.157	0.075
Fish to fish of show	3.017	0.19
Shrimp to fish off show	1.228	0.3
Fish to cuttlefish off show	3.791	0.005
Octopus to shark off show	2.555	0.040

Multiple post hoc comparisons (Bonferroni corrected) revealed where significances lay within the survey data arranged by source (i.e. UK aquarist etc.). For example, within the ‘fish to shark on show’ question significant differences lay between: UK aquarist and US aquarist (p = 0.032); UK aquarist and Zoo visitor (p <0.001) and UK aquarist and Aquarium visitor (p <0.001). A brief summary table has been made to indicate the significant comparisons found in S1 Table (in the supplementary materials) seen below in Table 5.

**Table 5:** Summary of the significant pairwise data.

On or Off Show	Scenario	Pair	p-value
On	Fish fed to shark	UK Aquarist and Zoo visitor	<0.01
On	Fish fed to shark	UK Aquarist and Aquarium visitor	<0.01
On	Fish fed to shark	UK Aquarist and US Aquarist	0.032
On	Fish fed to shark	UK Non-aquarist and Zoo visitor	0.02
On	Fish fed to shark	UK Non-aquarist and Aquarium visitor	0.023
On	Crab fed to cuttlefish	UK Aquarists and US Aquarists	0.02
On	Crab fed to cuttlefish	UK Aquarists and Zoo visitor	0.09
On	Crab fed to cuttlefish	UK Aquarists and Aquarium visitor	0.031
On	Crab fed to cuttlefish	US Aquarists and UK Non-aquarists	<0.01
On	Fish fed to fish	UK Aquarists and US Aquarists	<0.01
On	Fish fed to fish	UK Aquarists and Zoo visitors	0.016
On	Fish fed to fish	UK Aquarists and Aquarium visitors	0.005
On	Fish fed to fish	US Aquarists and UK Non-aquarists	0.001
On	Shrimp fed to fish	UK Aquarists and US Aquarists	0.013
On	Shrimp fed to fish	US Aquarists and UK Non-aquarists	<0.01
On	Fish fed to cuttlefish	UK Aquarists and US Aquarists	<0.01
On	Fish fed to cuttlefish	UK Aquarists and Zoo visitors	<0.01
On	Fish fed to cuttlefish	UK Aquarists and Aquarium visitors	0.003
On	Fish fed to cuttlefish	US Aquarists and UK Non-aquarists	0.002
On	Fish fed to cuttlefish	UK Non-aquarists and Zoo visitors	0.004
Off	Fish fed to shark	UK Aquarists and US Aquarists	0.001
Off	Fish fed to shark	UK Aquarists and Zoo visitors	<0.01
Off	Fish fed to shark	UK Aquarists and Aquarium visitors	<0.01
Off	Crab fed to cuttlefish	UK Aquarists and US Aquarists	<0.01
Off	Crab fed to cuttlefish	UK Aquarists and Zoo visitors	0.007
Off	Crab fed to cuttlefish	UK Aquarists and Aquarium visitors	0.009
Off	Crab fed to cuttlefish	US Aquarists and UK Non-aquarists	<0.01
Off	Crab fed to cuttlefish	UK Non-aquarists and Zoo visitors	0.005
Off	Crab fed to cuttlefish	UK Non-aquarist and Aquarium visitor	0.006
Off	Fish fed to fish	UK Aquarists and US Aquarists	<0.01
Off	Fish fed to fish	UK Aquarists and Aquarium visitors	0.003
Off	Fish fed to fish	US Aquarists and UK Non-aquarists	<0.01
Off	Fish fed to fish	US Aquarists an Zoo visitors	0.018
Off	Fish fed to fish	UK Non-aquarist and Aquarium visitor	0.016
Off	Shrimp fed to fish	UK Aquarists and US Aquarists	0.02
Off	Shrimp fed to fish	US Aquarists and UK Non-aquarists	0.001
Off	Shrimp fed to fish	US Aquarists and Zoo visitors	0.001
Off	Fish fed to cuttlefish	UK Aquarists and US Aquarists	<0.01
Off	Fish fed to cuttlefish	UK Aquarists and Zoo visitors	<0.01
Off	Fish fed to cuttlefish	UK Aquarists and Aquarium visitors	<0.01
Off	Fish fed to cuttlefish	US Aquarists and UK Non-aquarists	0.035
Off	Fish fed to cuttlefish	UK Non-aquarists and Zoo visitors	0.039
Off	Fish fed to cuttlefish	UK Non-aquarists and Aquarium visitors	0.017
Off	Octopus fed to shark	UK Aquarists and Zoo visitors	<0.01
Off	Octopus fed to shark	UK Aquarists and Aquarium visitors	0.001
Off	Octopus fed to shark	UK Non-Aquarists and Zoo visitors	0.005

It is noted that 20 out of the 22 significant results were using data from UK aquarists or UK non-aquarists as a comparison. See S1 Table in the supplementary material for a full list of significant and non-significant pairwise companions.

Table 6 details the differences between responses of participants by group and, as highlighted in bold, the significant differences in that response between species being used in the examples.

**Table 6:** Pairwise comparisons of on and off show results. The data failed parametric assumptions and Wilcoxon matched pairs were used to test significance.

Scenarios		On show		Off show		Wilcoxon	Test
UK Aquarists	N	Median	Std. Deviation	Median	Std. Deviation	Z	p
Octopus to shark	74	4.0	1.4	4.0	1.3	-3.407	**<0.001**
Crabs to cuttlefish	74	3.0	1.2	3.0	1.3	0.296	1.00
Fish to a cuttlefish	74	3.0	1.3	3.0	1.2	-0.46	1.00
Fish to sharks	74	3.0	1.4	3.0	1.3	-0.93	1.00
Fish to fish	74	3.0	1.2	3.0	1.2	0.463	1.00
Shrimp to fish	74	2.0	1.2	2.0	1.2	-2.426	**0.001**
**US Aquarists**							
Octopus to shark	36	4.0	1.1	4.0	1.2	1.278	0.164
Crabs to cuttlefish	36	2.0	0.8	2.0	0.9	0.958	1.00
Fish to a cuttlefish	36	3.0	0.8	3.0	0.9	-0.756	0.405
Fish to sharks	36	3.0	1.3	3.0	1.1	-0.333	0.940
Fish to fish	36	2.0	0.8	3.0	0.8	0.125	0.892
Shrimp to fish	36	2.0	0.7	2.0	1.0	-6.833	**<0.001**
**Non-aquarist UK professionals**							
Octopus to shark	54	4.0	1.4	4.0	1.4	-3.407	**0.017**
Fish to a cuttlefish	54	3.0	1.2	3.0	1.3	-0.46	0.951
Crabs to cuttlefish	54	2.5	1.2	3.0	1.3	0.296	0.693
Fish to fish	54	3.0	1.2	3.0	1.2	0.463	0.604
Fish to sharks	54	3.0	1.4	3.0	1.3	-0.93	0.902
Shrimp to fish	54	2.0	1.2	2.0	1.2	-2.065	**0.006**
**Just visited a zoo**							
Crabs to cuttlefish	50	2.0	0.8	3.0	1.0	0.418	0.595
Fish to a cuttlefish	50	2.0	0.8	3.0	1.3	-0.347	0.659
Fish to sharks	50	1.0	1.1	2.0	1.1	-1.929	**0.014**
Octopus to shark	50	3.0	1.2	3.0	1.4	-0.796	0312
Fish to fish	50	3.0	1.0	3.0	1.3	-1.041	0.186
Shrimp to fish	50	2.0	0.9	3.0	1.2	-2.388	**0.002**
**Just visited an aquarium**							
Crabs to cuttlefish	34	2.0	1.0	2.0	1	-0.471	0.618
Fish to a cuttlefish	34	2.0	1.1	2.0	1.1	0.500	0.597
Octopus to shark	34	3.0	1.1	3.0	1.3	0.44	0.963
Fish to sharks	34	1.0	1.1	2.0	1	-0.882	0.350
Fish to fish	34	4.0	0.9	4.0	1.1	-0.147	0.867
Shrimp to fish	34	1.5	1.2	4.0	1.1	-3.971	**<0.001**

## Discussion

The survey revealed differences in public perception based on where the participant is from, their background and the type of animal being used as prey. It is important to note here that Likert scales, despite allowing for a ‘neutral’ opinion, have been shown to be more reliable than a single ‘yes’ or ‘no’ answer and more appropriate to make inferences from [52]. The subjective interpretation of terms within a Likert scale could influence the results here; for example, ‘slightly unacceptable’ could be interpreted differently between individuals [53]. However, the questionnaire used simplistic wording to attempt to reduce misunderstandings, but these may still have occurred; especially where the researcher was not present to answer questions, i.e. via the online link.

The participants were chosen opportunistically, causing a potential bias in responses, which can be seen in Table 3. The main population is from the UK, of which there is a larger percentage of female participants from the ages of 18 to 34 years old. This may be contributed to by a larger percentage of women working in the animal welfare industry, yet this sample would still not be representative due to the large differences between groups.

**Fig 2:**
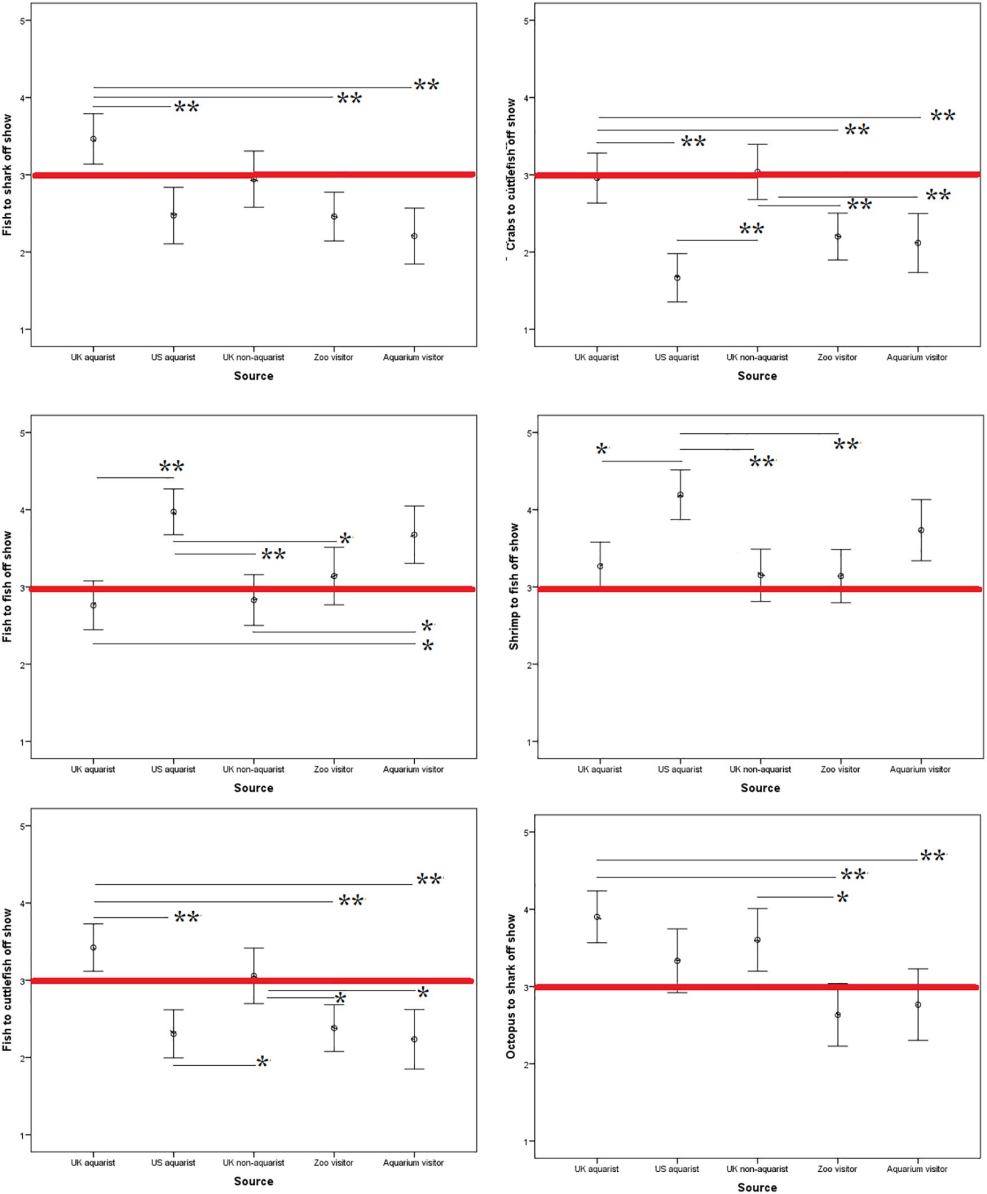
Mean survey scores by source (e.g. UK aquarist etc.) for all 12 questions asked regarding the acceptability of feeding various live aquatic animals to one another ‘off show’, i.e. not in view of the public. Liker scale (y-axis) ranged from 1 (least acceptable) to 5 (most acceptable), after recoding. The red line indicates the middle available score (i.e. ‘unsure’). Therefore, scores above the red line indicate that the practice is considered acceptable. * = p = <0.05 ** = p = <0.001.

Differences in opinion both between groups and species can be visualized in Figs 1 and 2 using plotted mean scores. A basic pattern can be seen whereby attraction visitors are less likely to find live prey feeding acceptable in most cases when compared to professionals.

### Feeding fish to shark

‘Fish’ is a relatively vague term that covers a variety of species, meaning that participants could be varied in their interpretation of this question. Visitors of the aquarium had seen a fish recently, but had no contact with a shark, potentially indicating why they were opposed to this scenario both on and off show if they had built empathy with fish. This theory would not, however, be supported by answers from UK professionals, who found this scenario most acceptable of all groups surveyed as they are likely to be familiar with fish; especially those working with them. This pattern emerges in many of the scenarios, both on and off show.

### Feeding crab to cuttlefish

The aquarium did not house any cuttlefish and only one species of crab (hermit crab) at the time of the survey, yet this scenario was significantly opposed by zoo and aquarium visitors as well as US professionals. UK professionals, again, were significantly more accepting of this.

Crab is a popular meat in the UK, especially in coastal regions (such as Paignton, where the surveys were taken), so it may be expected that this would influence scores of zoo and aquarium visitors into finding this more acceptable, yet the opposite is seen.

These findings may question whether an empathic response has been built from the learning style in zoos and aquariums that is generalized to aquatic life, a response which is individual to these establishments as UK professionals, who are likely to be educated well within their field, do not exhibit this.

### Feeding fish to fish

This scenario went against some of the previous patterns, with UK professionals being the most opposed when on show and US professionals and aquarium visitors finding it significantly acceptable if it is off show. This variation does raise, again, the reliability of this question if participants are considering a range of fish in their answers. Especially by using ‘fish’ both as prey and predator it could imply to a participant that the same species was being used on both roles, potentially eliciting concern of disease spread (such as a minor outbreak of Botulism in April 2017 in US).

### Feeding shrimp to fish

This scenario saw US consistently finding this scenario more acceptable, both on and off show. This may be expected due to the popularity of shrimp meat in the US. Aquarium visitors, however, also found this scenario more acceptable when off show. Whilst it could be argued that due to the lack of shrimp at the aquarium there was more of an empathic response to the predating fish in this question, when looking at responses to feeding ‘live crab to cuttlefish’, this did not seem to significantly impact the responses.

### Feeding fish to cuttlefish

This scenario saw UK professionals being significantly more accepting than any other group. The repetition of finding live prey feeding where a cuttlefish is the predator may stem from a higher empathic response from those who work with fish towards cuttlefish, as research about their higher cognitive abilities and electroreception is emerging. It would, however, then be expected that US professionals would follow this pattern, yet here it is seen that they, like the zoo and aquarium visitors, do not find this ethically acceptable; on or off show.

### Feeding octopus to shark

This scenario did evoke a different response, with responses being much less separated dependent on group. UK professionals were most opposed to this on show yet found it more acceptable when off show. Zoo and aquarium visitors found this more ethically acceptable than many other scenarios they had responded to.

This could stem from an excitement of seeing the hunting and feeding behavior and a recognition of ‘it is what happens on the wild’ that may be wanted within an education of the aquarium or zoo.

The responses from UK professionals finding this less acceptable than many other given scenarios within the survey may be, as assumed with cuttlefish, due to an empathic response to octopus. As cephalopods, octopi are regarded as more intelligent than many other aquatic species which may cause empathy from participants due to a presumed level of cognition closer to theirs and an attributed mental state. Fish, as a broad term, may be interpreted in many ways; all of which holding more emotional attachment of compassion than a shrimp or crab, which are commonly consumed in both the UK and US.

Similarly, the feeding behavior of sharks, whilst exciting to the public, may not be seen as an appropriate behavior for the public to view due to their representation in the media. This may be through reports of shark attacks and the subsequent pressures on local governments to prevent future attacks by means of public announcements [59]. This fear and negative association can be seen in a more subconscious suggestion in background music to televised shark scenes [60], which is a common accompaniment and can provoke fear in viewers.

### On and off show

The largest difference in responses seen was from UK professionals when feeding live octopus to sharks. It is considered that zoo and aquarium visitors as well as US professionals were, on average, less accepting of live prey feeding and therefore may not have changed their answers to even lower when the scenario was off-show.

Whilst zoo and aquarium visitors did score lower on the survey, the lack of change in response to live prey feeding on and off show may be due to the recent exposure to many of the species and feeling an involvement, therefore if the practices were to take place, participants may assume that they would not feel too differently whether they saw it or not. Despite a potential wariness of allowing children to see feeding, it seems to be more important to the visitors that they learn about ‘natural habits’ of the animals–including hunting and feeding. This could be a desire for seeing exciting things when they visit or from an educational point of view and understanding what happens; even teaching children there about how animals live.

### Professional participants

UK professionals were often in agreement on many scenarios, with UK non-aquarist professionals finding scenarios slightly more acceptable. US professionals, however, did not follow similar patterns often finding scenarios to be less ethically acceptable. These differences are not seen to be due to a separate variable as all professional surveys were completed online.

This is surprising, as it contradicts legislation in each country. It would be expected that UK professionals would adhere beliefs towards what the EU Directive has set out, and US professionals to be more willing to accept live prey feeding due to the lack of legislation directly prohibiting the act.

### Gender as an effect on ethical acceptability of live prey feeding

In previous studies [1, 2], females were more likely to find live prey feeding of terrestrial animals ‘slightly unacceptable’, yet the findings from this data did not reflect that, instead showing no significant differences between males and females. Due to a smaller sample size of males it is possible that this data is unreliable, however, there may also be explanations for the similarities.

The lack of difference in response based on gender varies from previous research from Ings [2], Cottle [1] and Ormandy and Schuppli [55]. Ormandy and Schuppli state that women are more likely to object to issues implicating animal rights as they are more likely to attribute mental states with animals. This may still be the case, however the mental state of the cuttlefish and sharks as predators may be a less imminent factor than it is with terrestrial animals.

The difference in fish and terrestrial animals with responses from the female demographic are defined by Panagiotarakou [56]. She states that whilst aretic (i.e. spiritual and totalitarianist), feminist-inspired ethics are suited to companion animal ethics they are not to endangered or ‘unlovable’ species. As discussed earlier, the decrease of emotion felt towards aquatic animals may be a reason why female opinions will be less predictable when discussing ‘unlovable’ animals.

It must also be considered that there are likely cultural changes from the results collected by Ings in 1997, both due to geography and the time difference. This may be one of the most significant reasons for the contrast in results based on gender.

### Experience of participant as an effect on the ethical acceptability of live prey feeding

Expectancy of differences between those that had recently visited a zoo or aquarium were that they would be more like professionals, due to zoos’ and aquariums’ long-term educational goals [57]. The data showed visitors that had just been to the zoo or aquarium were more opposed to live prey feeding than US aquarists and UK non-aquarists.

Potential reasons for this divide could be the immediate contact that participants had with the species. The survey was completed as zoo and aquarium visitors were leaving the establishments so, with help from species exposure and educational tools (such as posters, interactive games and talks), a short-term ‘ethic of care’ may have been created [58].

This same ethical opposition is seen less in professionals, especially within the UK. This may be due to a habituation to some species, meaning that this ‘ethic of care response’ is reduced. Due to the large variation of work completed in the profession, even just in the aquarist participants, it is unknown which other variables would affect this.

Previous studies [1, 2] have seen the demographic of participants with a higher education correlating with a higher acceptance of finding live prey feeding ethically acceptable. It is invalid to suggest that the UK and US professionals will all possess a higher level of education than zoo or aquarium participants, however it is much more likely that their education will be specific to animals; if not aquatic life particularly. This would imply that they are more familiar with welfare and husbandry regulations. This may be the reason that explains why there is such a difference in UK professionals and other groups’ responses.

## Conclusions

This study is the first of its kind to investigate public perceptions of live prey feeding in aquatic animals. It differs from previous work into terrestrial animals and those differences may help to understand the divide in perceptions of terrestrial and aquatic animals and why they exist.

Live prey feeding of aquatic animals; including vertebrates to vertebrates, invertebrates to invertebrates and invertebrates to vertebrates, was generally seen by participants as ‘somewhat acceptable’.

Significant differences appeared between UK and US professionals that contradicted the legislation in their country, yet visitors of zoos and aquariums were, on average, more opposed than any other group to live prey feeding. UK professionals most reflected the demographic found in previous papers of higher levels of education. This may be accurate, however without feedback from participants it is difficult to link these two variables.

Furthermore, gender differences were not seen as significantly as they were with regards to terrestrial animals; from studies by Ing and Cottle where females were more opposed to live prey feeding than males. Whilst there is not enough data to suggest that this difference is due to a reduced level of compassion, this gender similarity may be due to lowered levels of a compassion-like response (assuming these differences were caused by more compassion in female participants) to aquatics and invertebrates; possibly because of large phylogenetic differences.

It must be maintained, however, that similar, terrestrial studies were performed in 1997 and 2009. This time difference may account for the similarity of male and female responses as well as a geographical and cultural influence.

This paper highlights the general differences seen in this sample of participants dependant on their experiences, background and the species used in a scenario of live prey feeding. It may indicate why legislation for invertebrates and fish is less extensive when compared to their terrestrial counterparts when based on emotional responses towards them. Mostly, this paper demonstrates how differently ethical decisions are made when aquatic species are considered instead of terrestrial, limiting the generalisations that can be made about public perceptions to live prey feeding from existing work.

## Supporting information

S1 Survey The survey given to participants to complete. (DOCX)

S1 Table Multiple pairwise comparisons (Bonferroni corrected ANOVAs) for survey questions regarding the acceptability of feeding various live animals to one another, analysed by source i.e. UK aquarist etc. (DOCX)
