# Supplementary material for: Perception of the ethical acceptability of live prey feeding to aquatic species kept in captivity
Source: PLoS One. 2019 Aug 22;14(8):e0216777. doi: 10.1371/journal.pone.0216777 (PMC6705797; doi:10.1371/journal.pone.0216777)
Supplement: S1 Survey — (DOCX) [file pone.0216777.s001.docx]

S1: Survey (given to participants to complete:

1. What is your age?

1. 18-24 years
2. 25-34 years
3. 35-44 years
4. 45-54 years
5. 55-64 years
6. 65+ years

2. Which is your sex?

1. Female
2. Male

3. If you own any, what type/s of pets do you own?

1. Mammal
2. Bird
3. Reptile
4. Fish
5. Invertebrate

4. ‘Feeding live fish to sharks is ethically…’

1. Completely Acceptable
2. Slightly Acceptable
3. Neutral
4. Slightly Unacceptable

*Thank you for agreeing to participate in this research by completing this form. If you have any questions please feel free to ask me and please try to answer as honestly as you can.*

1. Completely Unacceptable

5. ‘Feeding live crabs to cuttlefish is ethically right’

1. Strongly Agree
2. Agree
3. Neutral
4. Disagree
5. Strongly Disagree

6. ‘Feeding live fish to another fish ethically wrong’

1. Strongly Agree
2. Agree
3. Neutral
4. Disagree
5. Strongly Disagree

7.  ‘Feeding live shrimp to fish is ethically…’

1. Completely Acceptable
2. Slightly Acceptable
3. Neutral
4. Slightly Unacceptable
5. Completely Unacceptable

8. ‘Feeding live fish to a cuttlefish is ethically right’

1. Strongly Agree
2. Agree
3. Neutral
4. Disagree
5. Strongly Disagree

9. ‘Feeding live octopus to a shark is ethically wrong’

1. Strongly Agree
2. Agree
3. Neutral
4. Disagree
5. Strongly Disagree

10. ‘Feeding live fish to sharks when there are no visitors is ethically right’

1. Strongly Agree
2. Agree
3. Neutral
4. Disagree
5. Strongly Disagree

11. ‘Feeding live crabs to cuttlefish when there are no visitors is ethically…’

1. Completely Acceptable
2. Slightly Acceptable
3. Neutral
4. Slightly Unacceptable
5. Completely Unacceptable

12. ‘Feeding live fish to another fish when there are no visitors is ethically wrong’

1. Strongly Agree
2. Agree
3. Neutral
4. Disagree
5. Strongly Disagree

13. ‘Feeding live shrimp to fish when there are no visitors is ethically wrong’

1. Strongly Agree
2. Agree
3. Neutral
4. Disagree
5. Strongly Disagree

14. ‘Feeding live fish to a cuttlefish when there are no visitors is ethically right’

1. Strongly Agree
2. Agree
3. Neutral
4. Disagree
5. Strongly Disagree

15. ‘Feeding live octopus to a shark when there are no visitors  is ethically…’

1. Completely Acceptable
2. Slightly Acceptable
3. Neutral
4. Slightly Unacceptable
5. Completely Unacceptable

16. ‘Simulating a live animal being fed to another animal is ethically right’

1. Strongly Agree
2. Agree
3. Neutral
4. Disagree
5. Strongly Disagree

17. ‘Pretending a dead animal is alive when feeding is ethically wrong’

1. Strongly Agree
2. Agree
3. Neutral
4. Disagree
5. Strongly Disagree
