# Supplementary material for: Perception of the ethical acceptability of live prey feeding to aquatic species kept in captivity
Source: PLoS One. 2019 Aug 22;14(8):e0216777. doi: 10.1371/journal.pone.0216777 (PMC6705797; doi:10.1371/journal.pone.0216777)
Supplement: S1 Table — (DOCX) [file pone.0216777.s002.docx]

S1 Table: Multiple pairwise comparisons (Bonferroni corrected ANOVAs) for survey questions regarding the acceptability of feeding various live animals to one another, analysed by source i.e UK aquarist etc

| Dependent Variable | | | Mean Difference (I-J) | Std. Error |  | Sig. | 95% Confidence Interval | |
| --- | --- | --- | --- | --- | --- | --- | --- | --- |
|  |  |  |  |  |  |  | Lower Bound | Upper Bound |
| Fish to shark on show | UK aquarist | US aquarist | .808^*^ | 0.272 |  | 0.032 | 0.04 | 1.58 |
|  |  | UK non-aquarist | 0.301 | 0.241 |  | 1.000 | -0.38 | 0.98 |
|  |  | Zoo visitor | 1.309^*^ | 0.245 |  | <0.01 | 0.61 | 2.00 |
|  |  | Aquarium visitor | 1.198^*^ | 0.277 |  | <0.001 | 0.41 | 1.98 |
|  | US aquarist | UK aquarist | -.808^*^ | 0.272 |  | 0.032 | -1.58 | -0.04 |
|  |  | UK non-aquarist | -0.507 | 0.287 |  | 0.784 | -1.32 | 0.31 |
|  |  | Zoo visitor | 0.501 | 0.290 |  | 0.855 | -0.32 | 1.32 |
|  |  | Aquarium visitor | 0.391 | 0.318 |  | 1.000 | -0.51 | 1.29 |
|  | UK non-aquarist | UK aquarist | -0.301 | 0.241 |  | 1.000 | -0.98 | 0.38 |
|  |  | US aquarist | 0.507 | 0.287 |  | 0.784 | -0.31 | 1.32 |
|  |  | Zoo visitor | 1.008^*^ | 0.262 |  | 0.002 | 0.27 | 1.75 |
|  |  | Aquarium visitor | .897^*^ | 0.292 |  | 0.023 | 0.07 | 1.72 |
|  | Zoo visitor | UK aquarist | -1.309^*^ | 0.245 |  | <0.001 | -2.00 | -0.61 |
|  |  | US aquarist | -0.501 | 0.290 |  | 0.855 | -1.32 | 0.32 |
|  |  | UK non-aquarist | -1.008^*^ | 0.262 |  | 0.002 | -1.75 | -0.27 |
|  |  | Aquarium visitor | -0.111 | 0.295 |  | 1.000 | -0.95 | 0.73 |
|  | Aquarium visitor | UK aquarist | -1.198^*^ | 0.277 |  | <0.001 | -1.98 | -0.41 |
|  |  | US aquarist | -0.391 | 0.318 |  | 1.000 | -1.29 | 0.51 |
|  |  | UK non-aquarist | -.897^*^ | 0.292 |  | 0.023 | -1.72 | -0.07 |
|  |  | Zoo visitor | 0.111 | 0.295 |  | 1.000 | -0.73 | 0.95 |
| Crabs to cuttlefish on show | UK aquarist | US aquarist | .859^*^ | 0.224 |  | 0.002 | 0.22 | 1.49 |
|  |  | UK non-aquarist | -0.235 | 0.199 |  | 1.000 | -0.80 | 0.33 |
|  |  | Zoo visitor | .679^*^ | 0.202 |  | 0.009 | 0.11 | 1.25 |
|  |  | Aquarium visitor | .683^*^ | 0.229 |  | 0.031 | 0.03 | 1.33 |
|  | US aquarist | UK aquarist | -.859^*^ | 0.224 |  | 0.002 | -1.49 | -0.22 |
|  |  | UK non-aquarist | -1.094^*^ | 0.237 |  | <0.001 | -1.77 | -0.42 |
|  |  | Zoo visitor | -0.180 | 0.240 |  | 1.000 | -0.86 | 0.50 |
|  |  | Aquarium visitor | -0.176 | 0.262 |  | 1.000 | -0.92 | 0.57 |
|  | UK non-aquarist | UK aquarist | 0.235 | 0.199 |  | 1.000 | -0.33 | 0.80 |
|  |  | US aquarist | 1.094^*^ | 0.237 |  | <0.001 | 0.42 | 1.77 |
|  |  | Zoo visitor | .914^*^ | 0.216 |  | <0.001 | 0.30 | 1.53 |
|  |  | Aquarium visitor | .918^*^ | 0.241 |  | 0.002 | 0.24 | 1.60 |
|  | Zoo visitor | UK aquarist | -.679^*^ | 0.202 |  | 0.009 | -1.25 | -0.11 |
|  |  | US aquarist | 0.180 | 0.240 |  | 1.000 | -0.50 | 0.86 |
|  |  | UK non-aquarist | -.914^*^ | 0.216 |  | <0.001 | -1.53 | -0.30 |
|  |  | Aquarium visitor | 0.004 | 0.244 |  | 1.000 | -0.69 | 0.69 |
|  | Aquarium visitor | UK aquarist | -.683^*^ | 0.229 |  | 0.031 | -1.33 | -0.03 |
|  |  | US aquarist | 0.176 | 0.262 |  | 1.000 | -0.57 | 0.92 |
|  |  | UK non-aquarist | -.918^*^ | 0.241 |  | 0.002 | -1.60 | -0.24 |
|  |  | Zoo visitor | -0.004 | 0.244 |  | 1.000 | -0.69 | 0.69 |
| Fish to fish on show | UK aquarist | US aquarist | -1.183^*^ | 0.230 |  | 0.000 | -1.84 | -0.53 |
|  |  | UK non-aquarist | -0.221 | 0.204 |  | 1.000 | -0.80 | 0.36 |
|  |  | Zoo visitor | -.663^*^ | 0.208 |  | 0.016 | -1.25 | -0.07 |
|  |  | Aquarium visitor | -.830^*^ | 0.235 |  | 0.005 | -1.50 | -0.17 |
|  | US aquarist | UK aquarist | 1.183^*^ | 0.230 |  | <0.001 | 0.53 | 1.84 |
|  |  | UK non-aquarist | .962^*^ | 0.243 |  | 0.001 | 0.27 | 1.65 |
|  |  | Zoo visitor | 0.520 | 0.246 |  | 0.356 | -0.18 | 1.22 |
|  |  | Aquarium visitor | 0.353 | 0.269 |  | 1.000 | -0.41 | 1.12 |
|  | UK non-aquarist | UK aquarist | 0.221 | 0.204 |  | 1.000 | -0.36 | 0.80 |
|  |  | US aquarist | -.962^*^ | 0.243 |  | 0.001 | -1.65 | -0.27 |
|  |  | Zoo visitor | -0.442 | 0.222 |  | 0.474 | -1.07 | 0.19 |
|  |  | Aquarium visitor | -0.609 | 0.247 |  | 0.145 | -1.31 | 0.09 |
|  | Zoo visitor | UK aquarist | .663^*^ | 0.208 |  | 0.016 | 0.07 | 1.25 |
|  |  | US aquarist | -0.520 | 0.246 |  | 0.356 | -1.22 | 0.18 |
|  |  | UK non-aquarist | 0.442 | 0.222 |  | 0.474 | -0.19 | 1.07 |
|  |  | Aquarium visitor | -0.167 | 0.250 |  | 1.000 | -0.88 | 0.54 |
|  | Aquarium visitor | UK aquarist | .830^*^ | 0.235 |  | 0.005 | 0.17 | 1.50 |
|  |  | US aquarist | -0.353 | 0.269 |  | 1.000 | -1.12 | 0.41 |
|  |  | UK non-aquarist | 0.609 | 0.247 |  | 0.145 | -0.09 | 1.31 |
|  |  | Zoo visitor | 0.167 | 0.250 |  | 1.000 | -0.54 | 0.88 |
| Shrimp to fish on show | UK aquarist | US aquarist | -.725^*^ | 0.222 |  | 0.013 | -1.35 | -0.10 |
|  |  | UK non-aquarist | 0.322 | 0.197 |  | 1.000 | -0.24 | 0.88 |
|  |  | Zoo visitor | -0.189 | 0.200 |  | 1.000 | -0.76 | 0.38 |
|  |  | Aquarium visitor | -0.110 | 0.226 |  | 1.000 | -0.75 | 0.53 |
|  | US aquarist | UK aquarist | .725^*^ | 0.222 |  | 0.013 | 0.10 | 1.35 |
|  |  | UK non-aquarist | 1.046^*^ | 0.234 |  | 0.000 | 0.38 | 1.71 |
|  |  | Zoo visitor | 0.536 | 0.237 |  | 0.247 | -0.14 | 1.21 |
|  |  | Aquarium visitor | 0.614 | 0.259 |  | 0.186 | -0.12 | 1.35 |
|  | UK non-aquarist | UK aquarist | -0.322 | 0.197 |  | 1.000 | -0.88 | 0.24 |
|  |  | US aquarist | -1.046^*^ | 0.234 |  | <0.001 | -1.71 | -0.38 |
|  |  | Zoo visitor | -0.511 | 0.214 |  | 0.177 | -1.12 | 0.10 |
|  |  | Aquarium visitor | -0.432 | 0.238 |  | 0.712 | -1.11 | 0.24 |
|  | Zoo visitor | UK aquarist | 0.189 | 0.200 |  | 1.000 | -0.38 | 0.76 |
|  |  | US aquarist | -0.536 | 0.237 |  | 0.247 | -1.21 | 0.14 |
|  |  | UK non-aquarist | 0.511 | 0.214 |  | 0.177 | -0.10 | 1.12 |
|  |  | Aquarium visitor | 0.079 | 0.241 |  | 1.000 | -0.60 | 0.76 |
|  | Aquarium visitor | UK aquarist | 0.110 | 0.226 |  | 1.000 | -0.53 | 0.75 |
|  |  | US aquarist | -0.614 | 0.259 |  | 0.186 | -1.35 | 0.12 |
|  |  | UK non-aquarist | 0.432 | 0.238 |  | 0.712 | -0.24 | 1.11 |
|  |  | Zoo visitor | -0.079 | 0.241 |  | 1.000 | -0.76 | 0.60 |
| Fish to Cuttlefish on show | UK aquarist | US aquarist | 1.072^*^ | 0.232 |  | <0.001 | 0.41 | 1.73 |
|  |  | UK non-aquarist | 0.155 | 0.206 |  | 1.000 | -0.43 | 0.74 |
|  |  | Zoo visitor | .955^*^ | 0.209 |  | <0.001 | 0.36 | 1.55 |
|  |  | Aquarium visitor | .861^*^ | 0.236 |  | 0.003 | 0.19 | 1.53 |
|  | US aquarist | UK aquarist | -1.072^*^ | 0.232 |  | <0.001 | -1.73 | -0.41 |
|  |  | UK non-aquarist | -.917^*^ | 0.245 |  | 0.002 | -1.61 | -0.22 |
|  |  | Zoo visitor | -0.117 | 0.248 |  | 1.000 | -0.82 | 0.59 |
|  |  | Aquarium visitor | -0.211 | 0.271 |  | 1.000 | -0.98 | 0.56 |
|  | UK non-aquarist | UK aquarist | -0.155 | 0.206 |  | 1.000 | -0.74 | 0.43 |
|  |  | US aquarist | .917^*^ | 0.245 |  | 0.002 | 0.22 | 1.61 |
|  |  | Zoo visitor | .800^*^ | 0.223 |  | 0.004 | 0.17 | 1.43 |
|  |  | Aquarium visitor | .706^*^ | 0.249 |  | 0.050 | 0.00 | 1.41 |
|  | Zoo visitor | UK aquarist | -.955^*^ | 0.209 |  | <0.001 | -1.55 | -0.36 |
|  |  | US aquarist | 0.117 | 0.248 |  | 1.000 | -0.59 | 0.82 |
|  |  | UK non-aquarist | -.800^*^ | 0.223 |  | 0.004 | -1.43 | -0.17 |
|  |  | Aquarium visitor | -0.094 | 0.252 |  | 1.000 | -0.81 | 0.62 |
|  | Aquarium visitor | UK aquarist | -.861^*^ | 0.236 |  | 0.003 | -1.53 | -0.19 |
|  |  | US aquarist | 0.211 | 0.271 |  | 1.000 | -0.56 | 0.98 |
|  |  | UK non-aquarist | -.706^*^ | 0.249 |  | 0.050 | -1.41 | 0.00 |
|  |  | Zoo visitor | 0.094 | 0.252 |  | 1.000 | -0.62 | 0.81 |
| Octopus to shark on show | UK aquarist | US aquarist | -0.327 | 0.274 |  | 1.000 | -1.10 | 0.45 |
|  |  | UK non-aquarist | -0.031 | 0.243 |  | 1.000 | -0.72 | 0.66 |
|  |  | Zoo visitor | -0.581 | 0.247 |  | 0.194 | -1.28 | 0.12 |
|  |  | Aquarium visitor | -0.521 | 0.279 |  | 0.629 | -1.31 | 0.27 |
|  | US aquarist | UK aquarist | 0.327 | 0.274 |  | 1.000 | -0.45 | 1.10 |
|  |  | UK non-aquarist | 0.296 | 0.289 |  | 1.000 | -0.52 | 1.11 |
|  |  | Zoo visitor | -0.254 | 0.292 |  | 1.000 | -1.08 | 0.57 |
|  |  | Aquarium visitor | -0.194 | 0.320 |  | 1.000 | -1.10 | 0.71 |
|  | UK non-aquarist | UK aquarist | 0.031 | 0.243 |  | 1.000 | -0.66 | 0.72 |
|  |  | US aquarist | -0.296 | 0.289 |  | 1.000 | -1.11 | 0.52 |
|  |  | Zoo visitor | -0.551 | 0.264 |  | 0.378 | -1.30 | 0.20 |
|  |  | Aquarium visitor | -0.491 | 0.294 |  | 0.963 | -1.32 | 0.34 |
|  | Zoo visitor | UK aquarist | 0.581 | 0.247 |  | 0.194 | -0.12 | 1.28 |
|  |  | US aquarist | 0.254 | 0.292 |  | 1.000 | -0.57 | 1.08 |
|  |  | UK non-aquarist | 0.551 | 0.264 |  | 0.378 | -0.20 | 1.30 |
|  |  | Aquarium visitor | 0.060 | 0.297 |  | 1.000 | -0.78 | 0.90 |
|  | Aquarium visitor | UK aquarist | 0.521 | 0.279 |  | 0.629 | -0.27 | 1.31 |
|  |  | US aquarist | 0.194 | 0.320 |  | 1.000 | -0.71 | 1.10 |
|  |  | UK non-aquarist | 0.491 | 0.294 |  | 0.963 | -0.34 | 1.32 |
|  |  | Zoo visitor | -0.060 | 0.297 |  | 1.000 | -0.90 | 0.78 |
| Fish to shark off show | UK aquarist | US aquarist | .993^*^ | 0.252 |  | 0.001 | 0.28 | 1.71 |
|  |  | UK non-aquarist | 0.521 | 0.223 |  | 0.203 | -0.11 | 1.15 |
|  |  | Zoo visitor | 1.005^*^ | 0.227 |  | <0.001 | 0.36 | 1.65 |
|  |  | Aquarium visitor | 1.259^*^ | 0.256 |  | <0.001 | 0.53 | 1.99 |
|  | US aquarist | UK aquarist | -.993^*^ | 0.252 |  | 0.001 | -1.71 | -0.28 |
|  |  | UK non-aquarist | -0.471 | 0.266 |  | 0.773 | -1.22 | 0.28 |
|  |  | Zoo visitor | 0.012 | 0.269 |  | 1.000 | -0.75 | 0.77 |
|  |  | Aquarium visitor | 0.266 | 0.294 |  | 1.000 | -0.57 | 1.10 |
|  | UK non-aquarist | UK aquarist | -0.521 | 0.223 |  | 0.203 | -1.15 | 0.11 |
|  |  | US aquarist | 0.471 | 0.266 |  | 0.773 | -0.28 | 1.22 |
|  |  | Zoo visitor | 0.483 | 0.242 |  | 0.473 | -0.20 | 1.17 |
|  |  | Aquarium visitor | 0.738 | 0.270 |  | 0.068 | -0.03 | 1.50 |
|  | Zoo visitor | UK aquarist | -1.005^*^ | 0.227 |  | 0.000 | -1.65 | -0.36 |
|  |  | US aquarist | -0.012 | 0.269 |  | 1.000 | -0.77 | 0.75 |
|  |  | UK non-aquarist | -0.483 | 0.242 |  | 0.473 | -1.17 | 0.20 |
|  |  | Aquarium visitor | 0.254 | 0.273 |  | 1.000 | -0.52 | 1.03 |
|  | Aquarium visitor | UK aquarist | -1.259^*^ | 0.256 |  | <0.001 | -1.99 | -0.53 |
|  |  | US aquarist | -0.266 | 0.294 |  | 1.000 | -1.10 | 0.57 |
|  |  | UK non-aquarist | -0.738 | 0.270 |  | 0.068 | -1.50 | 0.03 |
|  |  | Zoo visitor | -0.254 | 0.273 |  | 1.000 | -1.03 | 0.52 |
| Crab to cuttlefish off show | UK aquarist | US aquarist | 1.291^*^ | 0.246 |  | <0.001 | 0.60 | 1.99 |
|  |  | UK non-aquarist | -0.080 | 0.218 |  | 1.000 | -0.70 | 0.54 |
|  |  | Zoo visitor | .758^*^ | 0.222 |  | 0.007 | 0.13 | 1.39 |
|  |  | Aquarium visitor | .840^*^ | 0.250 |  | 0.009 | 0.13 | 1.55 |
|  | US aquarist | UK aquarist | -1.291^*^ | 0.246 |  | <0.001 | -1.99 | -0.60 |
|  |  | UK non-aquarist | -1.371^*^ | 0.259 |  | <0.001 | -2.11 | -0.64 |
|  |  | Zoo visitor | -0.533 | 0.262 |  | 0.432 | -1.28 | 0.21 |
|  |  | Aquarium visitor | -0.451 | 0.287 |  | 1.000 | -1.26 | 0.36 |
|  | UK non-aquarist | UK aquarist | 0.080 | 0.218 |  | 1.000 | -0.54 | 0.70 |
|  |  | US aquarist | 1.371^*^ | 0.259 |  | <0.001 | 0.64 | 2.11 |
|  |  | Zoo visitor | .838^*^ | 0.237 |  | 0.005 | 0.17 | 1.51 |
|  |  | Aquarium visitor | .920^*^ | 0.264 |  | 0.006 | 0.17 | 1.67 |
|  | Zoo visitor | UK aquarist | -.758^*^ | 0.222 |  | 0.007 | -1.39 | -0.13 |
|  |  | US aquarist | 0.533 | 0.262 |  | 0.432 | -0.21 | 1.28 |
|  |  | UK non-aquarist | -.838^*^ | 0.237 |  | 0.005 | -1.51 | -0.17 |
|  |  | Aquarium visitor | 0.082 | 0.267 |  | 1.000 | -0.67 | 0.84 |
|  | Aquarium visitor | UK aquarist | -.840^*^ | 0.250 |  | 0.009 | -1.55 | -0.13 |
|  |  | US aquarist | 0.451 | 0.287 |  | 1.000 | -0.36 | 1.26 |
|  |  | UK non-aquarist | -.920^*^ | 0.264 |  | 0.006 | -1.67 | -0.17 |
|  |  | Zoo visitor | -0.082 | 0.267 |  | 1.000 | -0.84 | 0.67 |
| Fish to fish off show | UK aquarist | US aquarist | -1.212^*^ | 0.246 |  | <0.001 | -1.91 | -0.51 |
|  |  | UK non-aquarist | -0.070 | 0.219 |  | 1.000 | -0.69 | 0.55 |
|  |  | Zoo visitor | -0.379 | 0.222 |  | 0.893 | -1.01 | 0.25 |
|  |  | Aquarium visitor | -.916^*^ | 0.251 |  | 0.003 | -1.63 | -0.20 |
|  | US aquarist | UK aquarist | 1.212^*^ | 0.246 |  | <0.001 | 0.51 | 1.91 |
|  |  | UK non-aquarist | 1.142^*^ | 0.260 |  | <0.001 | 0.40 | 1.88 |
|  |  | Zoo visitor | .832^*^ | 0.263 |  | 0.018 | 0.09 | 1.58 |
|  |  | Aquarium visitor | 0.296 | 0.288 |  | 1.000 | -0.52 | 1.11 |
|  | UK non-aquarist | UK aquarist | 0.070 | 0.219 |  | 1.000 | -0.55 | 0.69 |
|  |  | US aquarist | -1.142^*^ | 0.260 |  | <0.001 | -1.88 | -0.40 |
|  |  | Zoo visitor | -0.310 | 0.237 |  | 1.000 | -0.98 | 0.36 |
|  |  | Aquarium visitor | -.846^*^ | 0.265 |  | 0.016 | -1.60 | -0.10 |
|  | Zoo visitor | UK aquarist | 0.379 | 0.222 |  | 0.893 | -0.25 | 1.01 |
|  |  | US aquarist | -.832^*^ | 0.263 |  | 0.018 | -1.58 | -0.09 |
|  |  | UK non-aquarist | 0.310 | 0.237 |  | 1.000 | -0.36 | 0.98 |
|  |  | Aquarium visitor | -0.536 | 0.268 |  | 0.463 | -1.30 | 0.22 |
|  | Aquarium visitor | UK aquarist | .916^*^ | 0.251 |  | 0.003 | 0.20 | 1.63 |
|  |  | US aquarist | -0.296 | 0.288 |  | 1.000 | -1.11 | 0.52 |
|  |  | UK non-aquarist | .846^*^ | 0.265 |  | 0.016 | 0.10 | 1.60 |
|  |  | Zoo visitor | 0.536 | 0.268 |  | 0.463 | -0.22 | 1.30 |
| Shrimp to fish off show | UK aquarist | US aquarist | -.927^*^ | 0.247 |  | 0.002 | -1.63 | -0.23 |
|  |  | UK non-aquarist | 0.117 | 0.219 |  | 1.000 | -0.50 | 0.74 |
|  |  | Zoo visitor | 0.128 | 0.222 |  | 1.000 | -0.50 | 0.76 |
|  |  | Aquarium visitor | -0.468 | 0.251 |  | 0.640 | -1.18 | 0.24 |
|  | US aquarist | UK aquarist | .927^*^ | 0.247 |  | 0.002 | 0.23 | 1.63 |
|  |  | UK non-aquarist | 1.044^*^ | 0.260 |  | 0.001 | 0.31 | 1.78 |
|  |  | Zoo visitor | 1.054^*^ | 0.263 |  | 0.001 | 0.31 | 1.80 |
|  |  | Aquarium visitor | 0.459 | 0.288 |  | 1.000 | -0.36 | 1.28 |
|  | UK non-aquarist | UK aquarist | -0.117 | 0.219 |  | 1.000 | -0.74 | 0.50 |
|  |  | US aquarist | -1.044^*^ | 0.260 |  | 0.001 | -1.78 | -0.31 |
|  |  | Zoo visitor | 0.011 | 0.238 |  | 1.000 | -0.66 | 0.68 |
|  |  | Aquarium visitor | -0.584 | 0.265 |  | 0.283 | -1.33 | 0.17 |
|  | Zoo visitor | UK aquarist | -0.128 | 0.222 |  | 1.000 | -0.76 | 0.50 |
|  |  | US aquarist | -1.054^*^ | 0.263 |  | 0.001 | -1.80 | -0.31 |
|  |  | UK non-aquarist | -0.011 | 0.238 |  | 1.000 | -0.68 | 0.66 |
|  |  | Aquarium visitor | -0.595 | 0.268 |  | 0.272 | -1.35 | 0.16 |
|  | Aquarium visitor | UK aquarist | 0.468 | 0.251 |  | 0.640 | -0.24 | 1.18 |
|  |  | US aquarist | -0.459 | 0.288 |  | 1.000 | -1.28 | 0.36 |
|  |  | UK non-aquarist | 0.584 | 0.265 |  | 0.283 | -0.17 | 1.33 |
|  |  | Zoo visitor | 0.595 | 0.268 |  | 0.272 | -0.16 | 1.35 |
| Fish to cuttlefish off show | UK aquarist | US aquarist | 1.117^*^ | 0.241 |  | <0.001 | 0.43 | 1.80 |
|  |  | UK non-aquarist | 0.366 | 0.214 |  | 0.884 | -0.24 | 0.97 |
|  |  | Zoo visitor | 1.043^*^ | 0.217 |  | <0.001 | 0.43 | 1.66 |
|  |  | Aquarium visitor | 1.187^*^ | 0.246 |  | <0.001 | 0.49 | 1.88 |
|  | US aquarist | UK aquarist | -1.117^*^ | 0.241 |  | <0.001 | -1.80 | -0.43 |
|  |  | UK non-aquarist | -.751^*^ | 0.254 |  | 0.035 | -1.47 | -0.03 |
|  |  | Zoo visitor | -0.074 | 0.258 |  | 1.000 | -0.80 | 0.66 |
|  |  | Aquarium visitor | 0.070 | 0.282 |  | 1.000 | -0.73 | 0.87 |
|  | UK non-aquarist | UK aquarist | -0.366 | 0.214 |  | 0.884 | -0.97 | 0.24 |
|  |  | US aquarist | .751^*^ | 0.254 |  | 0.035 | 0.03 | 1.47 |
|  |  | Zoo visitor | .677^*^ | 0.232 |  | 0.039 | 0.02 | 1.33 |
|  |  | Aquarium visitor | .821^*^ | 0.259 |  | 0.017 | 0.09 | 1.55 |
|  | Zoo visitor | UK aquarist | -1.043^*^ | 0.217 |  | <0.001 | -1.66 | -0.43 |
|  |  | US aquarist | 0.074 | 0.258 |  | 1.000 | -0.66 | 0.80 |
|  |  | UK non-aquarist | -.677^*^ | 0.232 |  | 0.039 | -1.33 | -0.02 |
|  |  | Aquarium visitor | 0.145 | 0.262 |  | 1.000 | -0.60 | 0.89 |
|  | Aquarium visitor | UK aquarist | -1.187^*^ | 0.246 |  | <0.001 | -1.88 | -0.49 |
|  |  | US aquarist | -0.070 | 0.282 |  | 1.000 | -0.87 | 0.73 |
|  |  | UK non-aquarist | -.821^*^ | 0.259 |  | 0.017 | -1.55 | -0.09 |
|  |  | Zoo visitor | -0.145 | 0.262 |  | 1.000 | -0.89 | 0.60 |
| Octopus to shark off show | UK aquarist | US aquarist | 0.568 | 0.284 |  | 0.466 | -0.24 | 1.37 |
|  |  | UK non-aquarist | 0.298 | 0.252 |  | 1.000 | -0.42 | 1.01 |
|  |  | Zoo visitor | 1.269^*^ | 0.258 |  | <0.001 | 0.54 | 2.00 |
|  |  | Aquarium visitor | 1.137^*^ | 0.289 |  | 0.001 | 0.32 | 1.96 |
|  | US aquarist | UK aquarist | -0.568 | 0.284 |  | 0.466 | -1.37 | 0.24 |
|  |  | UK non-aquarist | -0.270 | 0.300 |  | 1.000 | -1.12 | 0.58 |
|  |  | Zoo visitor | 0.701 | 0.305 |  | 0.223 | -0.16 | 1.56 |
|  |  | Aquarium visitor | 0.569 | 0.332 |  | 0.880 | -0.37 | 1.51 |
|  | UK non-aquarist | UK aquarist | -0.298 | 0.252 |  | 1.000 | -1.01 | 0.42 |
|  |  | US aquarist | 0.270 | 0.300 |  | 1.000 | -0.58 | 1.12 |
|  |  | Zoo visitor | .971^*^ | 0.275 |  | 0.005 | 0.19 | 1.75 |
|  |  | Aquarium visitor | 0.839 | 0.305 |  | 0.064 | -0.03 | 1.70 |
|  | Zoo visitor | UK aquarist | -1.269^*^ | 0.258 |  | <0.001 | -2.00 | -0.54 |
|  |  | US aquarist | -0.701 | 0.305 |  | 0.223 | -1.56 | 0.16 |
|  |  | UK non-aquarist | -.971^*^ | 0.275 |  | 0.005 | -1.75 | -0.19 |
|  |  | Aquarium visitor | -0.132 | 0.310 |  | 1.000 | -1.01 | 0.75 |
